# Anaemia and long-term outcomes after surgical treatment of uterine fibroids: evidence from real-world data

**DOI:** 10.1007/s11239-026-03326-z

**Published:** 2026-05-23

**Authors:** Hani Essa, Dharani K. Hapangama, Ingeborg Welters, Gregory Y. H. Lip

**Affiliations:** 1 Institute of Life Course and Medical Sciences, Department of Cardiovascular and Metabolic Medicine, University of Liverpool, Liverpool, UK; 2 University Hospitals of Liverpool Group, Royal Liverpool University Hospital, Liverpool, UK; 3 Liverpool Centre for Cardiovascular Science, University of Liverpool, Liverpool John Moores University and Liverpool Heart & Chest Hospital, Liverpool, UK; 4 Department of Clinical Medicine, Aalborg University, Aalborg, Denmark; 5 Medical University of Bialystok, Bialystok, Poland

**Keywords:** Iron deficiency anaemia, Heavy menstrual bleeding

## Abstract

Uterine leiomyomas are the most common benign gynaecological tumours and a major cause of heavy menstrual bleeding, frequently resulting in iron deficiency anaemia. However, the long-term cardiovascular and thromboembolic consequences of fibroid-related anaemia remain poorly understood. We conducted a retrospective cohort study using the TriNetX health research network (~ 160 million patients). Women with uterine fibroids undergoing surgical management were identified and stratified by the presence or absence of anaemia within three years before surgery. Propensity score matching (1:1) was applied to balance demographics and comorbidities. Twelve-month outcomes included all-cause mortality, myocardial infarction, stroke, atrial fibrillation/flutter, heart failure, and venous thromboembolism. Risk ratios (RR) with 95% confidence intervals (CI) were calculated. From 83,319 eligible patients, 9,581 anaemic and 9,581 non-anaemic women were well matched. At one year, the presence of anaemia was associated with higher risks of mortality (RR 1.93, 95% CI 1.04–3.60, *p* = 0.03), stroke (RR 1.65, 1.05–2.60, *p* = 0.02), atrial fibrillation/flutter (RR 1.60, 1.03–2.48, *p* = 0.03), heart failure (RR 1.97, 1.43–2.70, *p* < 0.0001), and venous thromboembolism (RR 2.15, 1.67–2.80, *p* < 0.0001). No significant difference was observed for myocardial infarction (RR 1.44, 0.80–2.60, *p* = 0.22). The composite cardiovascular outcome was significantly elevated (HR 1.74, 95% CI 1.44–2.09, *p* < 0.0001). Preoperative anaemia in women undergoing fibroid surgery independently predicts adverse cardiovascular and thromboembolic events and nearly doubled 12-month mortality. Early recognition and optimisation of anaemia may improve clinical outcomes.

**Supplementary Information:**

The online version contains supplementary material available at 10.1007/s11239-026-03326-z.

## Introduction

Uterine fibroids (leiomyomas) are the most prevalent benign gynaecological tumours, with an estimated life-time incidence approaching 30–80% by the onset of menopause [1]. Although frequently asymptomatic, approximately half of affected women develop significant clinical manifestations, most commonly heavy menstrual bleeding (HMB), mass effect or a combination of both. Chronic HMB gradually depletes iron stores and reduces haemoglobin causing an iron-deficiency anaemia (IDA) [2]. IDA is linked to a wide range of adverse health outcomes, impacting both the physical and psychological well-being of women [3]. Fibroids, especially submucosal lesions are particularly associated with IDA [4].

Surgical management is commonly required for fibroid-related heavy menstrual bleeding, particularly when associated with significant anaemia [5]. Chronic anaemia has been linked to adverse cardiovascular and thromboembolic outcomes, including heart failure, arrhythmias, and stroke [6]. Even a small 1 g/dl reduction in haemoglobin is a well-established independent risk factor for cardiac morbidity and mortality [7]. Although haemoglobin correction may confer clinical benefit, chronic anaemia may result in partially irreversible cardiovascular changes [8]. Anaemia has also been linked to arrhythmias, notably atrial fibrillation (AF), and is increasingly recognised as a risk factor for ischaemic stroke in otherwise healthy premenopausal women [9, 10]. Moreover, through reactive thrombocytosis, anaemia may promote a hypercoagulable state and raise the risk of venous thromboembolism [11]. Taken together, these findings underscore that anaemia is a multisystem disorder with far-reaching clinical implications.

Despite growing evidence linking anaemia generally to adverse cardiovascular and thromboembolic outcomes, its long-term implications in women undergoing fibroid surgery, who are presumed to have a high prevalence of anaemia, remain largely unexplored. Most studies have focused on perioperative safety and short-term symptom relief, with limited attention to downstream cardiovascular sequelae. This study therefore aimed to fill the existing gap in the literature, and evaluate the long-term prognostic impact of anaemia in women undergoing operative management for uterine fibroids using a large, global federated real-world dataset.

## Methods

In this study, we sourced data from the TriNetX global health research network, which contains anonymized, real-world data from the electronic medical records of ~ 163 million individuals. This federated database represents 144 healthcare organizations in 14 countries. The platform allows for the construction of specific patient cohorts based on diagnoses, medications, and demographic information, providing a powerful tool for generating and validating research hypotheses. Data within the TriNetX platform are derived from routinely collected electronic health records and undergo standardisation and quality control processes prior to inclusion. As the platform provides access to de-identified, aggregated data without direct access to raw datasets, manual data cleaning, including handling of missing values or outliers, is not possible. Analyses are therefore conducted on available coded data, and patients with incomplete data for specific variables are excluded automatically from analyses involving those variables. The credibility of this network is underscored by its use in almost ~ 3300 peer-reviewed publications since 2018 (https://pubmed.ncbi.nlm.nih.gov/?term=trinetx).

All data handling was fully compliant with key data protection laws, including the Health Insurance Portability and Accountability Act (HIPAA) and the General Data Protection Regulation (GDPR). The TriNetX network protects patient data by performing all analyses locally at each partner healthcare organization (HCO). Only anonymized, aggregate results are shared, with no transfer of individual patient records. All contributing HCOs operate under formal data usage and publication agreements.

### Ethics statement

This study utilised anonymised data from the TriNetX global network. All participating healthcare organisations contribute data in compliance with their local ethical and legal requirements, including appropriate institutional approvals and patient consent where applicable.

The TriNetX platform provides access only to de-identified data, with no direct patient identifiers available to researchers. In accordance with institutional policies regarding the use of such data, formal ethical approval was not required.

### Study design

The search was conducted on September 24th, 2025, with predefined inclusion and exclusion criteria based on the relevant International Classification of Diseases, Tenth Revision, Clinical Modification codes (ICD-10) to create our cohort. We utilised codes designed to identify patients with fibroids undergoing operative management. All patients must be (1) diagnosed with Leiomyoma of the uterus (D25) and (2) undergoing operative management of their fibroids (ICD 10 (0uB93ZZ, 0u593zz, 0ub98zz, 0ub90zz, 0ub94zz, 0U598ZZ, 0Y594ZZ, 0U597ZZ, 0U590ZZ), SNOMED (446804002, 450559006), CPT (58140, 1008827, 58146, 58145, 1008871, 58546). The TriNetX database was searched with no date restrictions to allow us to capture the widest search results. Eligible patients were identified and then two cohorts created based on the presence of anaemia recorded in a patients medical records in the preceding 3 years prior to meeting our inclusion criteria.

### Statistical analysis

Data and outcomes were analyzed relative to the index event, the time window, and the predefined study endpoints. The index event was defined as the date on which a patient first met the study inclusion criteria (the index date). Follow-up was conducted over a 12-month time window beginning on the index date. Continuous variables with normal distribution were summarized as mean (standard deviation, SD).

Propensity score matching (PSM) was applied to minimize differences between the cohorts and address baseline variability. We selected variables that could influence outcomes to reduce the likelihood of confounding in our results. Patients with anemia were 1:1 propensity score matched to patients without anemia using logistic regression for age at index event, ethnicity (Black, White, and Asian), hypertension (I10), overweight and obesity (E66), asthma (J45), hyperlipidemia (E78.5), diabetes mellitus (E08–E13), ischemic heart disease (I20–I25), chronic kidney disease (N18), cerebrovascular disease including cerebral infarction (I63), alcohol-related disorders (F10), rheumatoid arthritis (M06), peripheral vascular disease (I73), gastroenteritis/colitis (K52), and chronic obstructive pulmonary disease (J44). These variables were chosen on the basis that they are established risk factors for cardiovascular disease/and or mortality or they were significantly different between both cohorts. The platform uses “greedy nearest-neighbour matching” with a calliper of 0.1 pooled SDs and difference between propensity scores of ≤ 0.1. Covariate balance between groups was assessed using strictly standardized mean differences. Any baseline characteristic with a standardized mean difference between cohorts of < 0.1 is considered well matched. Outcomes were evaluated over a 12-month follow-up period from the index event. Risk ratios (RR) with 95% confidence intervals (95% CI) were calculated for each outcome in the matched cohort.

Following PSM, risk ratios (RR) with 95% confidence intervals (CI) were calculated for one-year outcomes, including all-cause mortality (D; deceased), myocardial infarction (I21), stroke (I63; cerebral infarction), atrial fibrillation and flutter (I48), heart failure (I50), pulmonary embolism or deep vein thrombosis (I26, I80–I82) and a composite outcome encompassing all these events. Statistical analyses were conducted in R (version 4.3.1; R Foundation for Statistical Computing, Vienna, Austria) [12]. E-values were additionally calculated to assess the robustness of associations to potential unmeasured confounding. Statistical significance was set at *p* < 0.05.

## Results

A total of 83,319 patients were included before propensity score matching (PSM), comprising 9,581 (11.9%) patients with anaemia and 73,359 (88.1%) patients without anaemia. In the anaemia cohort (*n* = 9,581), the mean follow-up was 303.0 ± 120.3 days, with a median of 365 days (IQR: 4 days). The control cohort (*n* = 11,404) had a mean follow-up of 288.1 ± 133.1 days, with a median of 365 days (IQR: 116 days). To ensure comparability between groups, 1:1 PSM was performed, resulting in two matched cohorts of 9,581 patients each.

At baseline, patients with anaemia differed significantly from those without anaemia in both demographics and comorbidities, with strictly standardized mean differences (SSMDs) frequently exceeding 0.1, indicating meaningful imbalance. After PSM, the two cohorts were well balanced across all measured variables, with SSMDs generally below 0.05. Although some characteristics, such as diabetes mellitus, ischemic heart disease, and chronic kidney disease, remained statistically different (*p* < 0.05), the effect sizes were minimal (SSMD ≈ 0.03) and therefore below the threshold thought to be clinically meaningful [13]. Overall, PSM was effective in creating comparable groups of patients with and without anaemia. Baseline characteristics before and after matching are presented in Table 1. Supplementary Fig. 1 represents our PSM matching graph.

**Table 1:** Baseline characteristics of patients with anemia and without anemia before and after propensity score matching (PSM)

Variable	Before PSM			Post PSM		
	Anaemia cohort (9,581)	Control cohort (73,359)	*P*-Value, SSMD	Anaemia cohort (9,581)	Control cohort (9,581)	*P*-Value, SSMD
Demographics						
Age at Index	40.00 ± 8.80	40.40 ± 9.28	< 0.01, 0.05	40.00 ± 8.72	40.00 ± 8.96	0.64, 0.01
Black or African American	4,119 (42.67%)	13,879 (20.49%)	< 0.01, 0.49	4,067 (42.45%)	4,079 (42.57%)	0.86, 0.00
White	2,128 (22.05%)	17,325 (25.58%)	< 0.01, 0.08	2,120 (22.13%)	2,092 (21.84%)	0.63, 0.01
Unknown Ethnicity	2,033 (21.06%)	30,905 (45.63%)	< 0.01, 0.54	2,027 (21.16%)	1,969 (20.55%)	0.30, 0.01
Asian	1,359 (14.08%)	8,189 (12.09%)	< 0.01, 0.06	1,357 (14.16%)	1,379 (14.39%)	0.65, 0.01
Hispanic or Latino	960 (9.95%)	4,154 (6.13%)	< 0.01, 0.14	946 (9.87%)	946 (9.87%)	1.00, 0.00
Diagnoses						
Hypertension (I10)	2,005 (20.77%)	5,455 (8.05%)	< 0.01, 0.37	1,943 (20.28%)	1,915 (19.99%)	0.61, 0.01
Obesity (E66)	1,989 (20.61%)	5,176 (7.64%)	< 0.01, 0.38	1,935 (20.20%)	1,932 (20.17%)	0.96, 0.00
Asthma (J45)	1,097 (11.36%)	3,013 (4.45%)	< 0.01, 0.26	1,060 (11.06%)	1,050 (10.96%)	0.82, 0.00
Hyperlipidemia (E78.5)	837 (8.67%)	2,435 (3.60%)	< 0.01, 0.21	794 (8.29%)	787 (8.21%)	0.85, 0.00
Diabetes Mellitus (E08–E13)	733 (7.59%)	1,877 (2.77%)	< 0.01, 0.22	691 (7.21%)	611 (6.38%)	0.02, 0.03
Gastroenteritis/Colitis (K52)	469 (4.86%)	1,273 (1.88%)	< 0.01, 0.17	446 (4.66%)	439 (4.58%)	0.81, 0.00
Ischemic heart disease (I20–I25)	263 (2.73%)	602 (0.89%)	< 0.01, 0.14	238 (2.48%)	195 (2.04%)	0.04, 0.03
Vitamin B Deficiency (E53.8)	244 (2.53%)	308 (0.46%)	< 0.01, 0.17	209 (2.18%)	170 (1.77%)	0.04, 0.03
CKD (N18)	246 (2.55%)	346 (0.51%)	< 0.01, 0.17	206 (2.15%)	166 (1.73%)	0.04, 0.03
Alcohol related disorders (F10)	151 (1.56%)	286 (0.42%)	< 0.01, 0.12	135 (1.41%)	125 (1.31%)	0.53, 0.01
Rheumatoid arthritis (M06)	113 (1.17%)	322 (0.48%)	< 0.01, 0.08	109 (1.14%)	110 (1.15%)	0.95, 0.00
Peripheral vascular disease (I73)	105 (1.09%)	206 (0.30%)	< 0.01, 0.09	91 (0.95%)	68 (0.71%)	0.07, 0.03
Cerebral infarction (I63)	107 (1.11%)	155 (0.23%)	< 0.01, 0.11	89 (0.93%)	76 (0.79%)	0.31, 0.01
COPD (J44)	90 (0.93%)	236 (0.35%)	< 0.01, 0.07	77 (0.80%)	67 (0.70%)	0.40, 0.01

P values and SSMD (strictly standardized mean difference) are reported for each comparison. A P value ≤ 0.05 was considered statistically significant. PSM = Propensity Score Matching, SSMD = Strictly Standardized Mean Difference, CKD = Chronic Kidney Disease, COPD = Chronic Obstructive Pulmonary Disease

**Fig. 1:**
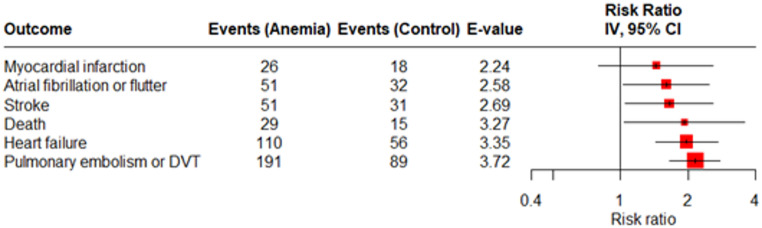
Forest plot of clinical outcomes at 12 months after operative management of uterine fibroids in patients with anaemia compared with matched controls. Risk ratios (RR) with 95% confidence intervals (CI) are shown for each outcome, alongside the number of events in each cohort and the corresponding E-values. Squares represent point estimates, with the size proportional to the weight of the estimate; horizontal lines indicate 95% CIs. An RR > 1 indicates higher risk in the anaemia cohort

**Fig. 2:**
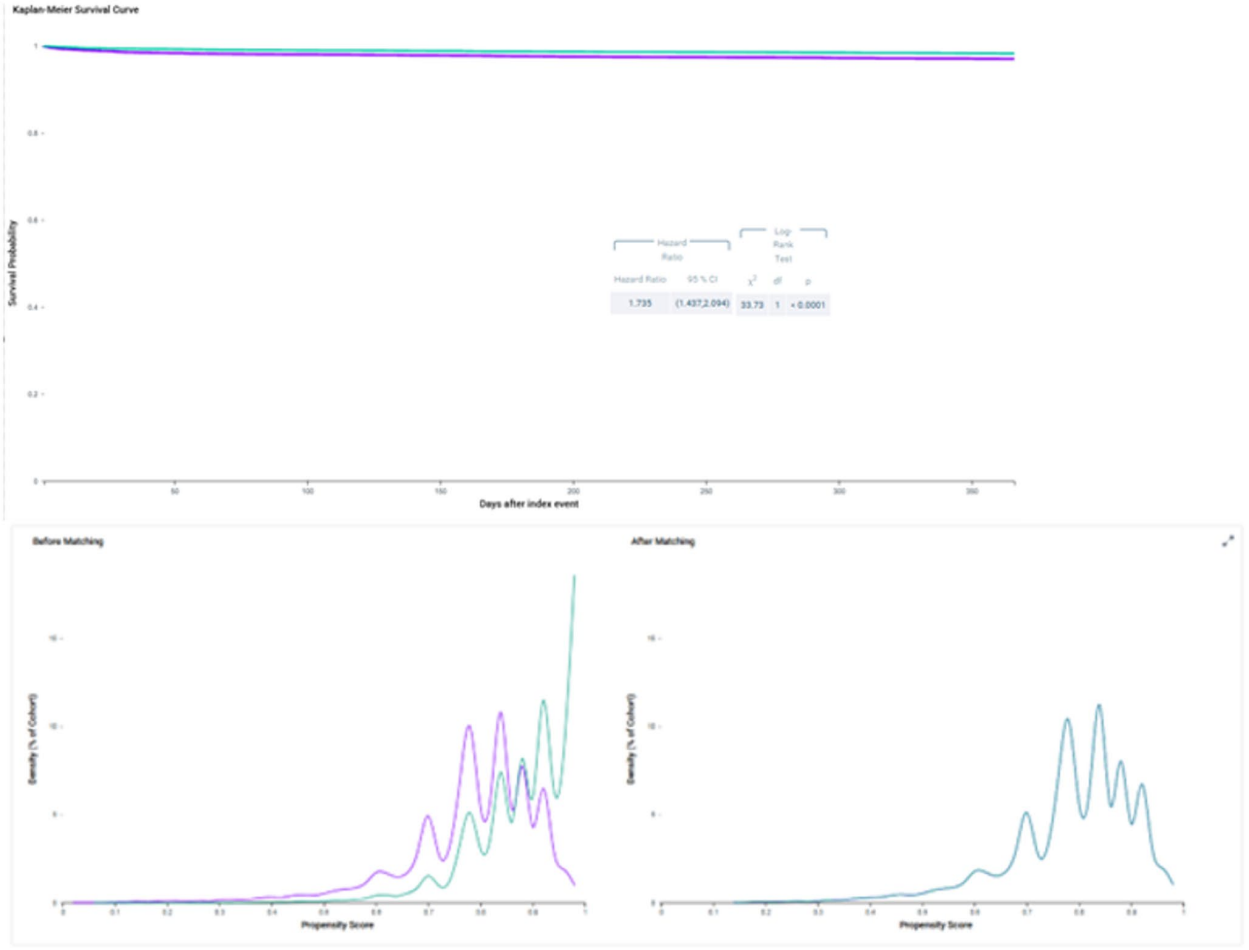
Kaplan–Meier survival curve for the composite outcome of myocardial infarction, atrial fibrillation/flutter, stroke, all-cause mortality, heart failure, and pulmonary embolism or deep vein thrombosis over 12 months following operative management of uterine fibroids in patients with anaemia compared with matched controls. The anaemia cohort demonstrated a significantly higher risk of the composite outcome (HR 1.74, 95% CI 1.44–2.09; log-rank χ² = 33.73, df = 1, *p* < 0.0001)

At 12 months following operative management of uterine fibroids (index event), patients with a background of anaemia experienced significantly higher mortality compared to controls (RR 1.93 [95% CI 1.04–3.60], *p* = 0.03), as well as an increased risk of stroke (RR 1.65 [95% CI 1.05–2.60], *p* = 0.02), atrial fibrillation or flutter (RR 1.60 [95% CI 1.03–2.48], *p* = 0.03), heart failure (RR 1.97 [95% CI 1.43–2.70], *p* < 0.0001), and venous thromboembolism (pulmonary embolism or deep vein thrombosis; RR 2.15 [95% CI 1.67–2.80], *p* < 0.0001) (Table 2). There was no statistically significant difference in the risk of myocardial infarction (RR 1.44 [95% CI 0.80–2.60], *p* = 0.22). The anaemia cohort demonstrated a significantly higher risk of the composite outcome (HR 1.74, 95% CI 1.44–2.09; log-rank χ² = 33.73, df = 1, *p* < 0.0001). Figure 1 presents a forest plot of these outcomes, and Kaplan–Meier curve of our composite outcomes is shown in Fig. 2.

**Table 2:** Clinical outcomes in patients with anaemia compared with matched controls

Outcome	Patients with outcome in anaemia cohort Vs control	Risk ratio	95% confidence interval	*P* Value	E-Value
Death	29 Vs 15	1.93	1.04–3.6	0.03	3.27
Myocardial infarction	26 Vs 18	1.44	0.8–2.6	0.22	2.24
Stroke	51 Vs 31	1.65	1.05–2.6	0.02	2.69
Atrial fibrillation and flutter	51 Vs 32	1.60	1.03–2.48	0.03	2.58
Heart failure	110 Vs 56	1.97	1.43–2.70	< 0.0001	3.35
Pulmonary embolism or deep vein thrombosis	191 Vs 89	2.15	1.67–2.80	< 0.0001	3.72

Results are expressed as risk ratios (RR) with 95% confidence intervals (CI) and corresponding *p* values. E-value represents the minimum strength of association, on the risk ratio scale, that an unmeasured confounder would need to have with both anemia and the outcome to fully explain away the observed association. Larger E-values suggest greater robustness of the association to unmeasured confounding

To enhance the robustness of our findings, we calculated E-values as a quantitative bias analysis to assess the potential impact of unidentified residual confounding [14]. Larger E-values suggest stronger evidence against unmeasured confounding as the sole explanation for the observed associations. Our results indicate that outcomes such as stroke (E-value 2.69), atrial fibrillation or flutter (2.58), heart failure (3.35), and venous thromboembolism (3.72) are relatively robust, as a strong unmeasured confounder would be required to fully account for these associations. In contrast, mortality (3.27) and myocardial infarction (2.24) demonstrated lower, though still moderate, robustness. Therefore, while residual confounding cannot be entirely ruled out, it is unlikely to be the sole explanation for the observed associations between anaemia and the major cardiovascular and thromboembolic endpoints.

## Discussion

In this large real-world cohort of ~ 19,000 women undergoing surgical management of uterine fibroids, our results demonstrate that patients with anaemia preoperatively exhibit significantly higher risk of death, stroke, atrial fibrillation and flutter, heart failure and venous thromboembolism compared to control patients without anaemia. There was no statistically significant increase in the risk of myocardial infarction, likely secondary to the low incidence of acute coronary events in a relatively young healthy female population.

Consistent with our results, a composite cardiovascular endpoint was significantly higher in the anaemia cohort. Our sensitivity analyses yielded moderately large E-values for key outcomes stroke, atrial arrhythmias, heart failure, and venous thromboembolism, indicating that an unmeasured confounder would need a relatively strong association (risk ratio on the order of 2.5–3.5) with both anaemia and the outcome to fully explain these associations. The Kaplan–Meier curves suggest an early clustering of events following surgery, raising the possibility that some outcomes may be driven by perioperative or early postoperative factors, including provoked events such as atrial arrhythmias, heart failure, and venous thromboembolism. Acute-on-chronic anaemia in the perioperative period may further contribute to this early risk. However, due to limitations of the dataset, we were unable to precisely determine the temporal relationship between surgery and event onset.

To the authors’ knowledge, this represents the largest study looking at cardiovascular outcomes in women with anaemia undergoing operative management of fibroids. These findings support heightened awareness and potentially enhanced perioperative optimization strategies in this population.

In our study, we found an incidence rate of anaemia of ~ 12%, this is slightly lower than the prevalence noted in a previous study by Murji et al.[15] in canada, who noted a prevalence of 23%. This discrepancy may be for several reasons. First, both studies relied on coded diagnoses which are liable to miscoding error. Second, their study was in a Canadian population, whilst ours included women from multiple different ethnic backgrounds, with potential differences in baseline health status and access to healthcare. In addition, ethnic differences in bleeding outcomes and sites have been reported [16]. Taken together, these difference highlight that whilst the exact prevalence may vary, anaemia remains a very common clinically significant comorbidity in women undergoing surgical management of fibroids.

The observed associations align with known pathophysiological consequences of chronic anaemia and iron deficiency. Severe IDA leads to compensatory hemodynamic changes including but not limited to physiological tachycardia, increased cardiac output and cardiac chamber dilation. This mechanistically likely explained the higher incidence of new heart failure and atrial arrhythmias in the anaemic cohort. Furthermore, chronic tissue hypoxia can predispose to cerebrovascular events [17] explaining the higher incidence of stroke. Taken together, the convergence of cardiovascular stressors (high-output cardiac strain, arrhythmogenic potential, and pro-thrombotic changes) in women with fibroid-related anaemia offers a biologically plausible explanation for their substantially worse 12-month outcomes relative to non-anaemic women.

Our findings also highlight uterine fibroids as a potential clinical model for studying the cardiovascular consequences of chronic anaemia. Unlike many older or multi-morbid populations in whom anaemia is prevalent, women with fibroids are typically younger and otherwise healthier. This provides a relatively “clean” setting in which the isolated effects of chronic anaemia on the cardiovascular system can be observed. The clustering of stroke, arrhythmias, heart failure, and venous thromboembolism in this cohort underscores the broader systemic risks associated with untreated anaemia. Future research may leverage this model to improve mechanistic understanding and to test targeted interventions, including iron optimisation, in advance of surgery.

Our findings have important clinical implications. Preoperative anaemia appears to identify a subgroup of women undergoing fibroid surgery who are at substantially higher risk of adverse cardiovascular and thromboembolic outcomes. This suggests that anaemia should not be viewed solely as a haematological abnormality, but as a potential marker of systemic physiological stress. In clinical practice, this supports the need for early recognition, thorough evaluation of underlying causes, and optimisation of haemoglobin levels prior to surgical intervention. There is growing evidence from other surgical populations supporting the correction of preoperative anaemia as part of patient blood management strategies. In both major abdominal and cardiac surgery, preoperative optimisation with iron therapy has been associated with increased haemoglobin levels and reduced perioperative transfusion requirements [18, 19], with some studies also suggesting improvements in postoperative recovery. For example, recent data from abdominal surgery cohorts demonstrate that intravenous iron therapy can effectively correct iron deficiency anaemia and reduce transfusion burden, while studies in cardiac surgery have shown that haemoglobin optimisation is associated with improved perioperative outcomes. Similarly, a randomized trial in neurosurgical patients (PICASA trial) reported that preoperative intravenous ferric carboxymaltose significantly reduced postoperative transfusion requirements [20]. Despite these encouraging findings, there remains a relative paucity of data specifically addressing the impact of preoperative anaemia correction in women undergoing fibroid surgery. Although correction of anaemia is recommended in clinical practice, the optimal preoperative haemoglobin target in this population remains unclear. Current guidelines generally aim for normalisation of haemoglobin levels (e.g., ≥ 120 g/L in women), but further studies are needed to determine whether specific thresholds are associated with improved cardiovascular outcomes in this setting [21]. Incorporating anaemia into perioperative risk stratification models may help guide monitoring intensity and preventative strategies in this population. Future studies should investigate whether preoperative correction of anaemia can mitigate this excess risk. Prospective cohort or interventional studies with detailed haemoglobin trajectories, iron indices, and echocardiographic data are needed to establish causal mechanisms and guide perioperative optimization strategies in this population.

### Limitations

Several limitations of this study warrant consideration. First, this is a retrospective analysis based on data extracted from an electronic database in the form of ICD-10 codes, without access to individual patient charts. This raises the possibility of incomplete or inaccurate coding, since the data are primarily collected for routine care and billing. Second, all statistical analyses were conducted using the TriNetX built-in platform, which restricts user access to raw data and does not permit fully customizable multivariable Cox regression analyses within propensity score–matched cohorts, limiting our ability to perform additional post-matching adjustment. Third, we are unable to establish the precise timing of events, only whether they occurred within the observation window. Moreover, while we report median follow-up duration, the database does not provide granular details such as the exact number of patients remaining at study end. Another limitation relates to the large size of the TriNetX network: although this increases statistical power, it also raises the likelihood of detecting statistically significant but clinically trivial associations. Additionally, we did not have granular information on fibroid disease severity (e.g. fibroid size/number, extent of blood loss) or details of the surgical procedure (myomectomy vs. hysterectomy, intraoperative blood loss, transfusions received). These factors could influence post-operative outcomes. An additional limitation relates to the nature of routinely collected EHR data, which are primarily generated for clinical care and administrative or billing purposes. As such, the indication for laboratory testing, including complete blood count measurement, cannot be determined. Consequently, the presence of anaemia may reflect a heterogeneous group of underlying conditions and not solely fibroid-related bleeding. Furthermore, given the relatively young mean age of the study population (~ 40 years), alternative causes of abnormal uterine bleeding and anaemia, for example adenomyosis, may be present but are not fully captured within the dataset. These factors introduce the potential for indication bias and residual confounding, which may influence the observed associations. Additionally, given the observational design, causal inferences cannot be drawn, and unmeasured confounders may have influenced the findings. Additionally, Kaplan–Meier analyses generated within the TriNetX platform have notable limitations that affect interpretation. The system operates largely as a proprietary platform with limited transparency. Researchers cannot influence data cleaning, censoring methods, or underlying modelling assumptions. Follow-up is highly variable, and it is not possible to reliably distinguish true censoring from limited follow-up. For example, patients who die, leave the network, or discontinue care are indistinguishable in the dataset. In addition, the graphical output lacks key analytical features, including confidence intervals, censoring indicators, and detailed statistical parameters. Finally, while our study demonstrates the prognostic significance of preoperative anaemia in women undergoing fibroid surgery, we were unable to directly compare the prevalence of anaemia to women undergoing other surgical procedures (e.g., cholecystectomy) within a similar age range. Such comparisons would help clarify whether the burden of anaemia is uniquely elevated in fibroid patients or reflective of background prevalence in women of reproductive age. For these reasons, we consider our findings to be hypothesis-generating and emphasize the need for confirmatory studies.

## Conclusion

Our study suggests that preoperative anaemia in women undergoing surgery for uterine fibroids may be associated with an increased risk of adverse cardiovascular and thromboembolic outcomes, including higher mortality within one year. However, given the observational design and inherent limitations of real-world data, these findings should be interpreted with caution and do not establish causality. Further prospective studies are needed to determine whether optimisation of anaemia can reduce these risks.

## Electronic Supplementary Material

Below is the link to the electronic supplementary material.

Supplementary Material 1

## Data Availability

The data used is part of the TriNetX network and our results may be re-created on the network using the methodology described.

## Abbreviations

CI: Confidence Interval
CPT: Current Procedural Terminology
GDPR: General Data Protection Regulation
HCO: Healthcare Organization
HIPAA: Health Insurance Portability and Accountability Act
HR: Hazard Ratio
ICD: 10–International Classification of Diseases, Tenth Revision
IDA: Iron Deficiency Anaemia
IQR: Interquartile Range
KM: Kaplan–Meier
MI: Myocardial Infarction
PSM: Propensity Score Matching
RR: Risk Ratio
SD: Standard Deviation
SNOMED: Systematized Nomenclature of Medicine–Clinical Terms
SSMD: Strictly Standardized Mean Difference
TriNetX: TriNetX Global Health Research Network
VTE: Venous Thromboembolism

## Declarations

### Generative AI Use

Artificial intelligence tools were used solely to assist in identifying spelling and grammatical errors during the manuscript review process. No AI tools were used for data analysis, interpretation, or drafting of the scientific content.
