# Supplementary material for: Anaemia and long-term outcomes after surgical treatment of uterine fibroids: evidence from real-world data
Source: J Thromb Thrombolysis. 2026 May 23;59(6):1659–66. doi: 10.1007/s11239-026-03326-z (PMC13447403; doi:10.1007/s11239-026-03326-z)
Supplement: Supplementary file 1 [file 11239_2026_3326_MOESM1_ESM.docx]

Supplementary figure 1 - **Propensity score density plots before and after matching.**
The left panel shows the distribution of estimated propensity scores for the treatment group (teal) and the control group (purple) prior to matching, demonstrating clear differences in score distributions. The right panel shows the distributions after propensity score matching, where the two groups are well aligned, indicating improved covariate balance between treatment and control groups.
